# Digital elevation models of the sea-ice surface from airborne laser scanning during MOSAiC

**DOI:** 10.1038/s41597-023-02565-6

**Published:** 2023-10-20

**Authors:** Nils Hutter, Stefan Hendricks, Arttu Jutila, Robert Ricker, Luisa von Albedyll, Gerit Birnbaum, Christian Haas

**Affiliations:** 1 Cooperative Institute for Climate, Ocean and Ecosystem Studies, University of Washington, Seattle, WA 98105 USA; 2 Alfred-Wegener-Institut, Helmholtz-Zentrum für Polar- und Meeresforschung, Bremerhaven, 27570 Germany; 3 NORCE Norwegian Research Centre, Tromsø, 9019 Norway; 4 Institute of Environmental Physics, University of Bremen, Bremen, 28334 Germany; 5 Present Address: Finnish Meteorological Institute, Helsinki, 00560 Finland

**Keywords:** Cryospheric science, Physical oceanography

## Abstract

Airborne laser scanners (ALS) are used to map the sea-ice surface at sub-meter resolution. We conducted 64 flights over the Arctic sea ice between September 2019 and September 2020 during the Multidisciplinary drifting Observatory for the Study of Arctic Climate (MOSAiC) expedition to measure sea-ice surface elevation. The flights ranged from repeated, local-scale 5 × 5 km^2^ floe grid surveys to regional-scale transects more than 100 km long. We provide data at different processing levels: geolocated elevation point clouds and gridded segments of elevation and freeboard with a spatial resolution of 0.5 m. The latter product is corrected for atmospheric backscatter, sea-ice drift, and offset in elevation due to degraded INS/GNSS solutions > 85° N. For floe grid surveys, all data are combined to merged two-dimensional elevation maps. Other provided parameters include laser reflectance and echo width. The presented data offer a unique possibility to study the temporal evolution, spatial distribution, and variability of the snow and sea-ice surface and their properties in addition to validating satellite products.

## Background & Summary

Airborne laser altimeters have been used to measure the state of the Arctic sea-ice surface for several decades starting with single-beam laser profilers in the 1960s^1^. As technology has advanced, airborne laser scanners (ALS) that provide elevations in a two-dimensional swath under the aircraft have been used more frequently, e.g. as part of the CryoSat Validation Experiment^2^ (CryoVEx, European Space Agency, since 2003), Operation IceBridge^3^ (National Aeronautics and Space Administration, 2009–2019), and IceBird^4^ (Alfred Wegener Institute, since 2009) campaigns. With precise knowledge of the aircraft’s position and attitude, the range data from the ALS are converted to ellipsoidal elevation of the snow and sea-ice surface that gives information about sea-ice conditions such as roughness or the size and spatial distributions of surface features. Ellipsoidal surface elevation can be further converted to freeboard, i.e. the height of the air–snow or air–ice interface above the local water level, which is used routinely to retrieve sea-ice thickness from satellite laser altimetry or Ka-band radar altimetry data^5^. Therefore, ALS data are also used for validation of various satellite products and often combined with airborne radar systems^6–11^. However, high-resolution ALS mapping to capture the seasonal evolution of the same ice over several months has not been done before.

The year-long Multidisciplinary drifting Observatory for the Study of Arctic Climate (MOSAiC) expedition of the research vessel Polarstern^12^, moored to an ice floe, aimed to study the seasonal evolution of physical snow and sea-ice properties and processes. Over a large part of an annual cycle from October 2019 to July 2020, the expedition drifted passively with the sea ice along the Transpolar Drift from north of the Laptev Sea, across the central Arctic Ocean, and towards the Fram Strait. For logistical reasons, this part of the expedition was split into four legs. For the fifth and final leg in August–September 2020, Polarstern relocated and moored to a new floe close to the North Pole. Detailed measurements spanned across spatial scales from local, centimeter-scale in the Central Observatory (CO,  <5 km from Polarstern) to regional scale of up to few tens of kilometers in the Distributed Network (DN,  <50 km from Polarstern) of autonomous measurement platforms. For more detailed description of the expedition and the research related to the snow and sea-ice cover, we refer the reader to the overview article by Nicolaus *et al*.^13^.

**Fig. 1:**
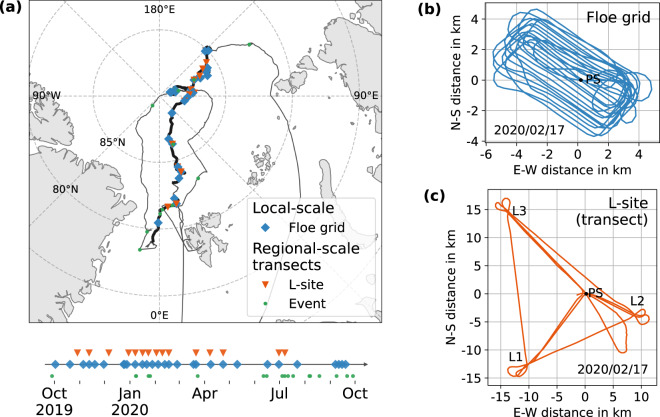
(**a**) Overview of all ALS surveys performed during MOSAiC. The path on the map shows the route of Polarstern (PS) where the thicker line marks the passive drift. The different markers represent the survey type of each flight. Typical flight patterns for the floe grid and L-site flights are shown in panels (**b**) and (**c**), respectively. Note the different scale of distance axes.

During the five legs of the MOSAiC expedition, we carried out a total of 64 helicopter flights with a primary focus on documenting the temporal evolution of the spatial distribution and variability of the snow and sea-ice surface and their properties with an ALS (Fig. 1a). There were two survey types: (1) floe grids, i.e. local-scale surveys in grid pattern over the CO and the surrounding sea-ice floes within the approximately 5 × 5 km^2^ survey area (Fig. 1b); and (2) transects, i.e. regional-scale surveys with up to approximately 100 km long straight sections. The transect flights often followed a triangular pattern between the three large sensor suite nodes (L-sites) of the DN and Polarstern (Fig. 1c). In addition, there were opportunistic event surveys, e.g. of sea-ice dynamics such as newly formed leads and pressure ridges or of melt ponds, that fall into either of the two categories. We aimed to conduct repeated local and regional-scale measurements in approximately weekly intervals.

**Fig. 2:**
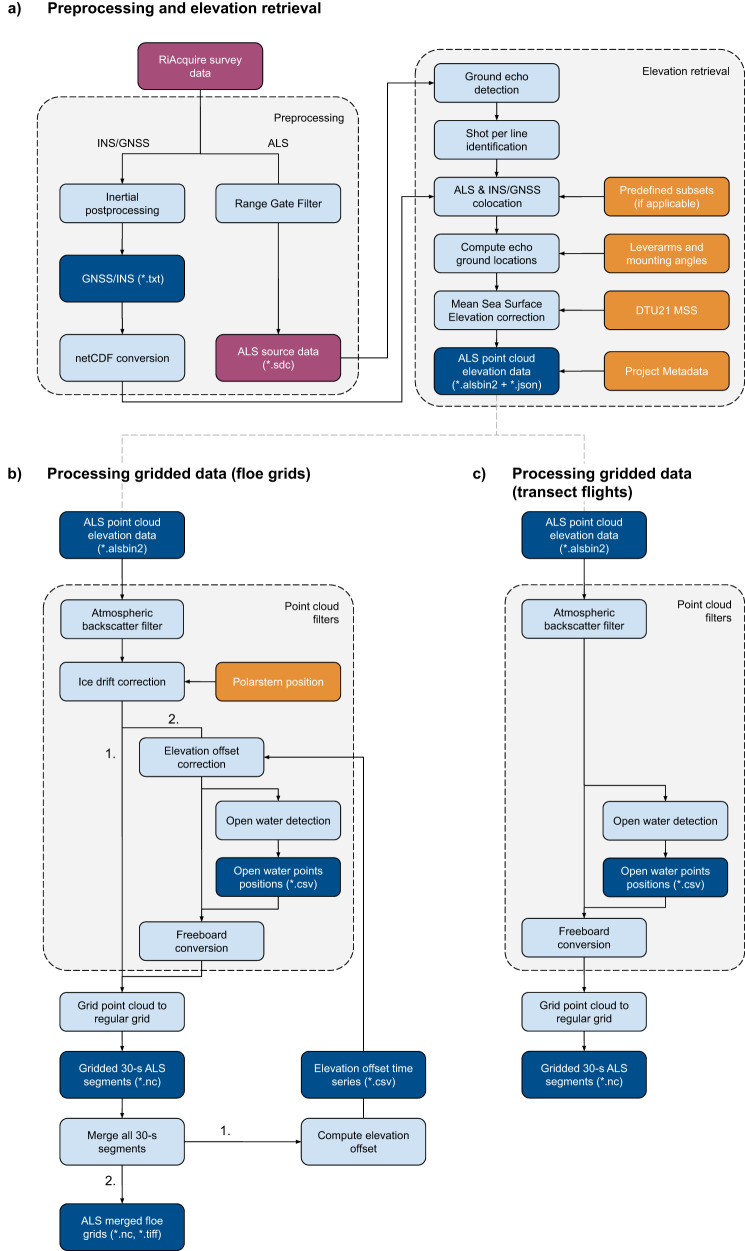
Flow chart of the processing pipeline from raw sensor data to elevation point clouds (**a**), and elevation point clouds to final gridded data products ((**b**) for floe grid flights and (**c**) for transect flights). All published data records are highlighted in dark blue. Raw sensor data and data products of intermediate processing steps that are planned to be released at a later point in time are marked in purple. Auxiliary information used in the processing are given in orange.

In brief, our processing workflow for the data resulting from those flights was the following. First, we converted the range measurements from the ALS into geolocated sea-ice and snow surface elevation point clouds (Fig. 2a). In further processing steps (Fig. 2c), we split the point cloud data into 30-second segments, from which we filtered out possible atmospheric backscatter. Then, we performed freeboard conversion using an automated open water detection scheme before projecting the point cloud data onto a regular 0.5-m grid. For the local-scale grid-pattern flights, we applied additional corrections for ice drift and elevation offset before freeboard conversion and gridding (Fig. 2b). Here, we present four data products with varying processing levels: helicopter INS/GNSS data, surface elevation point clouds, gridded segments, and merged floe grids.

## Methods

### Sensor set-up and data collection

**Fig. 3:**
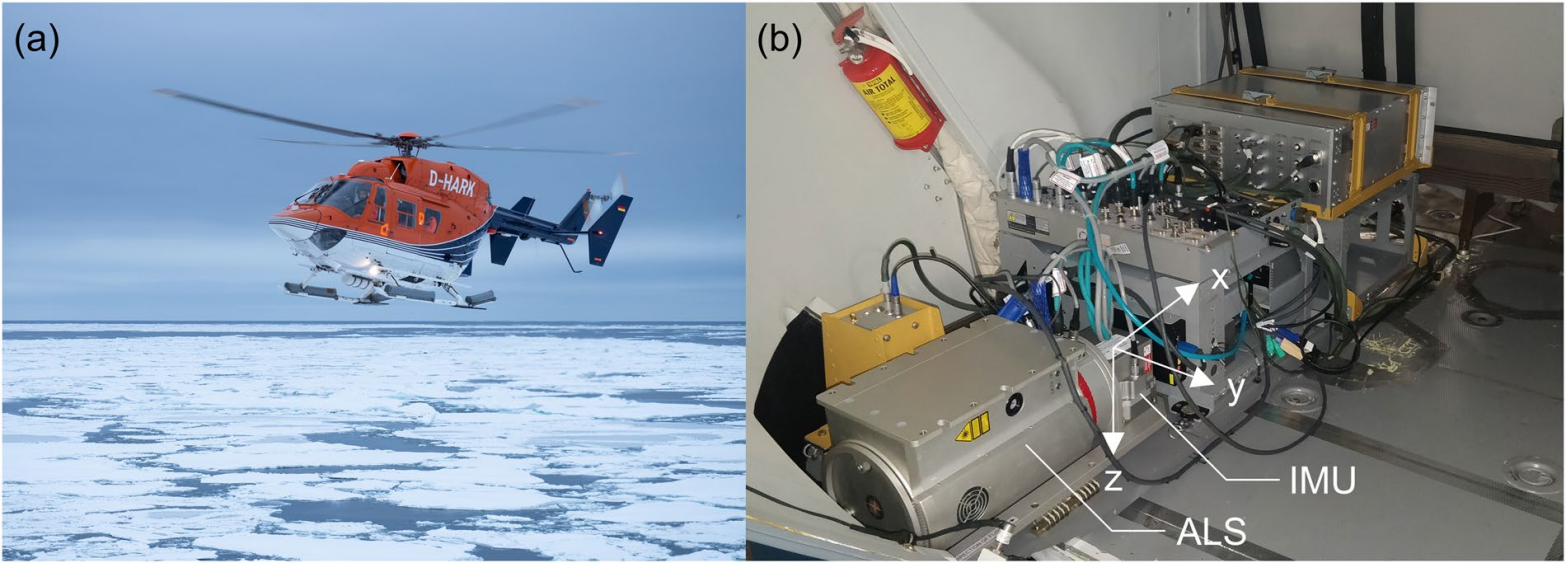
The measurement set-up. (**a**) The helicopter onboard Polarstern used as the measurement platform. Photo credit: Alfred-Wegener-Institut/Jan Rohde (CC-BY 4.0). (**b**) Configuration of the Riegl VQ-580 airborne laser scanner (ALS) and the inertial measurement unit (IMU) in the cargo compartment of the helicopter. The aircraft coordinate system is defined by the x-axis in flight direction, y-axis to the right side of the aircraft and z-axis in nadir direction shown with white arrows. The laser scanner is looking down at nadir through a cutout in the rear door. Photo credit: Alfred-Wegener-Institut/Stefan Hendricks.

The near-infrared (wavelength 1064 nm), line-scanning Riegl VQ-580 airborne laser scanner^14–16^ (ALS), providing ranging data in an accuracy and precision of 25 mm, was mounted on an exchangeable sensor platform together with other instruments and data acquisition units in the cargo compartment of an Airbus Helicopter MBB-BK 117 C-1 on board Polarstern (Fig. 3). An external inertial measurement unit (IMU-57) was integrated on the same mounting plate as part of the combined global navigation satellite system (GNSS), using data from Global Positioning System (GPS), Global Navigation Satellite System (GLONASS), and Beidou satellites, and inertial navigation system (INS) AP60-Air^17^ from Applanix. The IMU received input signals from two AV39 GNSS antennae installed on the forward and aft cowlings on top of the main cabin of the helicopter. The position and attitude data were recorded with a rate of up to 200 Hz. The position of the sensors in the aircraft coordinate system and the rotation with respect to their own reference frame are documented in Table 1. The aircraft coordinate system is defined by the x-axis in flight direction, y-axis to the right side of the aircraft and z-axis in nadir direction (Fig. 3b). The mounting angles *γ*, *β*, *α* describe the rotation around the x, y, z axes, respectively. Due to a broken power supply, the ALS sensor of leg 1 was replaced with another sensor of the same make and model for the remaining legs 2–5. The replacement changed the mounting angle of the ALS.

**Table 1:** Positions (x, y, z) in meters and mounting angles (*γ*, *β*, *α*) in degrees of the airborne laser scanner (ALS), the inertial measurement unit (IMU), and the front and rear global navigation satellite system (GNSS) antennae in the aircraft reference frame.

	Lever arm (x, y, z) m	Orientation (*γ*, *β*, *α*) degrees
**ALS**	( − 0.35, 0.0, 0.02)	(0.0, 0.0, 180.0) leg 1 ( − 0.32, 0.0, 180.0) leg 2–5
**IMU**	(0.0, 0.0, 0.0)	(0, 0, − 90)
**GNSS Rear (Primary)**	( − 0.580, 0.282, − 1.640)	
**GNSS Front (Seconary)**	(2.620, 0.197, − 1.540)	

During the flights, we aimed at recording data with the highest areal coverage possible while maintaining a high point density. During the polar night, the minimum and nominal survey altitude was 1000 ft ( ≈ 300 m) due to safety reasons. For some flights in summer, we adjusted the flight altitude to 500 ft ( ≈ 150 m) or even 200 ft ( ≈ 60 m) to acquire data in higher resolution or to adapt to the changing cloud base height. Table 2 summarizes the survey parameters.

**Table 2:** Summary of the key survey parameters used during MOSAiC.

Parameter	Nominal	High resolution
Altitude (above ground level)	1000 ft ( ≈ 300 m)	
Ground speed	90 kn ( ≈ 45 m s^−1^)	60 kn ( ≈ 30 m s^−1^)
Field of view	60^°^	
Laser pulse repetition rate	300 kHz	380 kHz
Field-of-view resolution	0.0555^°^	0.0395^°^
Swath width	351.953 m	
Scan rate	127.01 Hz	125.71 Hz
Line distance	0.3240 m	0.2455 m
Point distance	0.3239 m	0.2457 m
Point density	9.52 pts m^−2^	16.58 pts m^−2^

Altitude, ground speed, field of view, and laser pulse repetition rate are user-defined parameters. Field-of-view resolution, swath width, scan rate, line and point distance, as well as point density are calculated and reported by the operating software RiAcquire provided by Riegl.

Throughout the MOSAiC expedition, a number of other scientific instruments were operated simultaneously on the same helicopter platform, such as a thermal infrared camera^18^ in winter and in summer visible range RGB^19^ and hyperspectral (visible and near-infrared) cameras, an infrared radiation pyrometer, as well as upward and downward looking pyranometers.

#### INS alignment procedures

Airborne inertial navigation systems require an attitude alignment before the start of the measurements. However, this alignment is difficult with gyroscopic information alone close to the North Pole with a low horizontal component of the Earth rotation rate^20^. Ship-based studies up to a latitude below 81° N (i.e. far south of our main study region) have shown that INS gyroscopic data inaccuracy leads to rapidly increasing heading error as a function of decreasing distance to the North Pole^21^. There are two possibilities for achieving INS alignment. During static alignment, the aircraft is not moving and the INS/GNSS system determines its heading with data from the two GNSS receivers. Dynamic alignment uses motion of the aircraft, preferably an S-shaped curve, to determine the heading based on GNSS observations of the moving aircraft and known locations (lever arms) of the GNSS receivers. System tests on the helicopter deck with ground power demonstrated that static alignment could not be reached even after more than 1 hour at approximately 86° N. Long waiting times before take-off on the helicopter deck with air temperatures well below −10°C, the lower limit of the operation temperature range of the ALS sensor^14^, were also not practical. We therefore followed dynamic alignment procedures for INS initialization directly after take-off.

### Preprocessing

An overview of the preprocessing steps is presented in Fig. 2a. We used the SDCImport 2.3 software provided by the ALS manufacturer Riegl to convert the raw RiAcquire survey data files into the extracted data format (ALS source data) with a fixed multiple time around (MTA) zone and a manually adjusted range gate to filter out outliers with erroneous elevations. This preprocessing step also included a built-in, simple echo classification procedure of reflection targets that distinguishes between ground and atmospheric backscatter sources based on the number of targets (echo signals), echo width, and distance to previous echo signals. Only ground reflection targets are relevant for this study.

#### INS/GNSS postprocessing

Obtaining the position and attitude of the helicopter with high precision requires inertial postprocessing and data fusion of the accelerations and angular rates measured by the INS as well as all incoming GNSS data measured by the two GPS/GNSS antennae. We used the POSPac Mobile Mapping Suite (MMS) 8.3 software by Applanix in precise point positioning (PPP) mode. This mode allows obtaining centimeter-scale precision only based on precise ephemerides of the GNSS satellites. An external GPS/GNSS reference station is not required and would have been difficult to maintain on the moving sea ice or ship with the nearest land more than 1000 km away. Using a Kalman filter, the software provides a solution based on the observations of the two GNSS receivers, the IMU, the lever arms and the orientation of the sensors in the aircraft reference frame. The result is high-resolution position and attitude of the origin of the aircraft reference frame, defined by the position of the IMU at a temporal resolution of 200 Hz. Quality control output of POSPac MMS shows a vertical uncertainty of 10 cm, a horizontal uncertainty of 5 cm and an angular uncertainty of 0.2 arc minutes (0.003 degrees). As the final step of preprocessing, we converted the merged position and attitude data file into separate GNSS and INS Network Common Data Format (netCDF) files using a transcription tool Sea Convert written on Interactive Data Language (IDL).

Unfortunately, at the time of writing, the PPP solutions could not be generated for approximately 67 % of the GNSS and INS data, all located at very high latitudes of > 85° N. For the affected surveys, only the real-time navigation (RTNav) solution is available. The RTNav solution is generated by the IMU with a resolution of 10 Hz and used as real-time input for the ALS control software. Compared to PPP, the RTNav solution includes GNSS data at lower accuracy and precision as well as drift of the attitude angles. The level of the drift depends on the actual flight path and regular turns help to minimize INS drift.

Troubleshooting with help from the Applanix customer support team indicated that the Kalman filter could not converge to a solution, likely due to a low signal-to-noise ratio of the IMU data caused by in-flight vibrations. But there are likely other aggravating factors leading to the failure of the PPP postprocessing. Kalman filters for inertial processing depend on good knowledge of the initial state^20^ and data before take-off and after landing were not recorded to minimize helicopter operations on the ship. Also GNSS data quality in the polar regions suffers from visibility issues at lower elevations of the sky combined with shadowing of satellites by parts of the aircraft. When the signal is received, ranging biases may occur from ionospheric activity.

Based on our analysis (see section Degraded INS/GNSS solutions in Technical Validation), we assumed that the RTNav horizontal positioning and attitude information were still adequate for the purpose of processing of the ALS data, but the vertical position uncertainty was insufficient for surveys with only RTNav solution available unless further corrections were made. Based on an extensive evaluation of the processed freeboard data (see section Technical Evaluation), we conclude that without correcting for the elevation offset with overlapping swaths the freeboard uncertainty is unreasonable high and we therefore refrain from providing freeboard estimates for all transect flights with RTNav solutions. In the remainder of the paper, we will use the following terminology: the degraded GNSS quality for RTNav solutions leads directly to elevation errors manifested in surface elevation undulations. We refer to the differences between the observed and the true surface elevation as elevation offset. As part of the processing, we computed an elevation offset correction where sufficient overlapping swaths were available, i.e. for the floe grid surveys. We computed and applied this correction regardless to both RTNav and PPP derived elevations.

### Elevation retrieval

Figure 2a shows the processing steps taken to retrieve elevation from the ALS data. This was done using a customized IDL software tool als_level1b_seaice. First, we detected ground echoes by choosing the echo with the largest range value for each laser shot and filtering out atmospheric backscatter targets using the classification performed in preprocessing. We then sorted the remaining final ground echoes into scan lines. Next, we colocated the ALS data with the GNSS and INS data to compute the corresponding surface elevations above the WGS84 ellipsoid using the information on the angle and range measurements of the ALS, the lever arms and mounting angles of the sensor, as well as the position and attitude of the aircraft. Finally, we referenced the surface elevations to the gridded 1-minute resolution mean sea surface (MSS) height product of the Technical University of Denmark (DTU21 MSS^22^). We then combined each echo with the corresponding reflectance and echo width (full width at half maximum) information. We stored the resulting point cloud data in custom binary file format *.alsbin2 and nominally split the data into 5-minute along-track segments. Each file is accompanied by a JavaScript Object Notation (*.json) metadata file. We provide a tool to parse the binary files as part of the python-based awi-als-toolbox package.

### Processing of regular-gridded data

We interpolated the binary point cloud ALS data to a regular grid with a spatial resolution of 0.5 m to compress the very dense binary data and to facilitate the analysis of spatial characteristics of the ALS data. The basic steps of the gridding were (1) reading in the point cloud data and splitting them into 30-second segments; (2) depending on survey type and season, applying various filters on the point cloud data such as atmospheric backscatter filter, ice drift correction, open water detection (all described in detail in the sections below); (3) linearly interpolating all point data to a regular grid with fixed resolution in a common reference coordinate system using the same projection; (4) computing the freeboard from elevation measurements; and (5) for floe grid surveys, mapping all 30-s segments into one file of the entire flight and correct the elevation for any offsets caused by uncertain vertical RTNav GNSS positioning. Depending on the survey type, the order of these basic steps was changed or some steps were repeated. A detailed outline of all processing steps for each survey type is given in Fig. 2b,c.

#### Atmospheric backscatter filter

The near-infrared laser pulse of the ALS is not only reflected by the water and ice on the surface but also by liquid or ice clouds, haze, or diamond dust that are in the path of sight. Therefore, low-altitude clouds or fog that were present during the flight lead to point measurements with very high elevations. To not mix those high elevation points from clouds in the interpolation step with elevation signal from the ice/ocean surface, we filtered point measurements related to clouds. In doing so, we computed for each 30-s segment the modes of the elevation distribution and eliminated all elevation measurements that exceeded a threshold of ±20 m around the lowest peak. Even though the actual elevation variations of the ice–ocean surface within the survey were lower than 20 m, we set a higher threshold to account for the elevation offset caused by the low quality RTNav GNSS altitude solutions. Note that all clouds observed during the surveys were at altitudes higher than 20 m.

#### Ice drift correction

All point elevation measurements were referenced to their geographical position at the time of data acquisition, i.e. to the position of the laser scanner at that time. During the average survey flight time of approximately 2 h, the ice constantly drifted. Krumpen *et al*.^23^ report that the average drift speed during MOSAiC was 8.52 km d^−1^, which corresponds to a drift distance of 710 m during a two-hour survey. To combine all elevation measurements into one map, we needed to correct for the relative ice drift between the acquisition of the point measurements similar to a previous study^24^ mapping the sea-ice surface in larger 2-d areas in drifting ice. For the commonly performed transect type surveys with little to no overlap such a correction is not required. We used Polarstern’s position and heading data recorded with the high performance INS Motion Sensor Hydrins 1^25^ at a frequency of 10 min for this correction, taking advantage of the fact that the ship was rigidly anchored to and drifting with the ice. The data were downloaded automatically using the AWI DASHBOARD Data Web Service portal (https://dashboard.awi.de/data-xxl/) application programming interface (API). Based on Polarstern’s position and heading record, we determined both the translational and rotational motion of the ice pack between the acquisition time and the reference time of the flight (mid-point of the flight) and corrected the position of the elevation point measurement accordingly. We did this computation for all elevation point measurements in the same reference projection and coordinate system, which is a stereographic projection centered around Polarstern’s position at the reference time. We rotated the projection with the heading of Polarstern at the reference time to align the x-axis with the longitudinal axis of Polarstern, where the positive x-axis is towards the bow. Using only Polarstern’s position as a reference, we assumed that the ice drift in the entire survey area was homogeneous, i.e. no deformation took place during the flight. As this assumption is likely violated with increasing distance away from Polarstern, we applied the ice drift correction only to the floe grid flights in the vicinity of Polarstern (approximately 5 km) and not to the much more distant transect flights.

#### Open water detection

To retrieve freeboard from the elevation measurements, we needed to identify open water points to use them as tie points for interpolating the sea surface height in the survey area. In this section, we present an automated open water detection algorithm that runs on the ALS point cloud data.

The principle of the open water detection is to identify open water by its unique signatures in both the reflectance of the laser pulse and the elevation, similar to the approach used by Kwok *et al*.^26^ for data from the Airborne Topographic Mapper (ATM) system on the IceBridge mission. Liquid water reflects the laser with little scatter. The direction of the reflected laser pulse is determined by the orientation of the open water surface. In calm conditions with a smooth ocean surface, the laser pulse is reflected back to the scanner in nadir and we observe a high reflectance, an effect called glint. If winds are roughening the water surface by inducing small waves, the laser pulse is reflected away from the laser scanner and very low reflectance values are measured, if at all. In summary, the open water surface is characterized by strong deviations (both minima and maxima) from the background reflectance of snow and ice that both are scattering the laser pulse more strongly. In addition to this signal in reflectance, the open water surface also shows a minimum in the elevation measurements as their freeboard is zero.

**Fig. 4:**
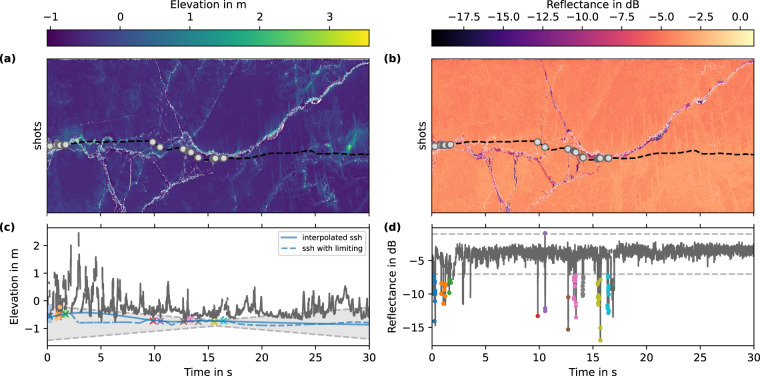
Illustration of open water detection algorithm for a 30-s segment from the survey on March 23rd, 2020: (**a**) elevation, (**b**) reflectance, (**c**) elevation in nadir beam, and (**d**) reflectance in nadir beam. The positions of nadir measurements are shown as the black dashed line in (**a**) and (**b**). The detected clusters of open water points are marked with gray circles in (**a**) and (**b**). In (**c**) and (**d**), open water points are shown in colored dots, where all points belonging to the same cluster are identified by the same color. The lower and upper thresholds for both elevation and reflectance are indicated by the gray dotted lines and the shaded area in (**c**) highlights the range of elevation that potential open water points could have.

The open water detection was applied to the 30-s segments of the point cloud data. We extracted all nadir measurements using the information on the roll angle of the helicopter and the angular resolution of the laser scanner (note that by doing so we assumed a constant zero pitch angle over the 30-s segment). First, we filtered all nadir measurements for points that show low elevation values. In doing so, we computed the global elevation minimum of the 30-s segment ${h}_{min}=h\left({t}_{min}\right)$. To account for the temporally varying offset in elevation, we defined the following filter criterion at time *t*:

1 $$\left|h\left(t\right)-{h}_{min}\right|\le \Delta {h}_{offset}\frac{\left|t-{t}_{min}\right|}{30\,{\rm{s}}}+{\sigma }_{h},$$

where Δ*h*_*offset*_ is the maximum difference in elevation offset within a 30-s segment, and *σ*_*h*_ is the uncertainty in the elevation compared to its local surrounding. Table 3 provides the values of these parameters dependent on season and GNSS quality available for the flight. Note that we assumed that the elevation offset varies linearly with time over the 30-s segment in Eq. (1). All elevation values that fulfilled this criterion are illustrated by the gray shaded area in Fig. 4c. The filter criterion for the reflectance *r* was given by:

2 $$rt-\bar{r} > {r}_{t},\,{\rm{with}}\,{r}_{t}=3\,\mathrm{dB}\,,$$

where $\bar{r}$ is the mean reflectance of the 30-s segment, *r*_*t*_ is a threshold here determined to be 3 dB. All point measurements that fulfilled both criteria in Eqs. (1) and (2) were identified as open water. In the last step, we collected all open water points that belonged to the same lead into clusters. By averaging all open water points within a cluster, we could reduce the uncertainty in the elevation of the open water surface within an individual lead as we averaged over multiple elevation measurements. All open water points that were not farther apart than 0.2 s from each other were assigned to the same cluster. At the nominal flight speed of 45 m s^−1^, this corresponded to a maximum spacing of 9 m between open water points belonging to the same cluster.

**Table 3:** Parameters used in the freeboard conversion depending on season (melt vs. freezing conditions) and quality of GNSS solution.

Parameter	winter		summer
GNSS solution	RTNav	PPP	PPP
reflectance threshold *r*_*t*_	3 dB		
elevation uncertainty *σ*_*h*_	5 cm	5 cm	2.5 cm
max drift in elevation Δ*h*_*offset*_	1 m	0.2 m	0.1 m

PPP stands for precise point positioning and RTNav for real-time navigation solutions.

#### Freeboard conversion

We interpolated the sea surface height from the measured elevations of the open water points in leads to determine freeboard for all elevation measurements. Depending on the survey type, we applied different interpolation routines: for transect surveys, we applied a 1-d spline interpolation as commonly done for this kind of data^4,27^. Here, we used a smoothing factor of 0.03 and the timestamp of each measurement as the coordinate for the interpolation. For the floe grid surveys, where we obtained a larger 2-d spatial coverage, we used a 2-d Radial Basis Function (RBF)^28^ interpolation with linear kernel and smoothing factor of 10 that used the ice drift corrected position of each measurement for the interpolation. This 2-d interpolation method also takes into account open water points detected in adjacent swaths while producing a smooth sea surface height. Note that the smoothing factors of both interpolators differ, since the interpolation used different coordinates for the different survey types (space for floe grids and time for transects). Nevertheless, we chose them to represent roughly the same length scale. An example of the interpolated sea surface height is shown in Fig. 4c. Once we determined the interpolator based on the detected open water clusters, we computed the freeboard by subtracting the interpolated sea surface height from each elevation point measurement. This point-wise freeboard data were then gridded in the same manner as the other point-cloud data (elevation, reflectance, and echo width).

The high latitude position of Polarstern and the close pack ice with few lead openings posed a major challenge to determine reliable sea level heights. The low quality RTNav GNSS solutions could lead to strong undulations in the elevation between far spaced open water points that resulted in both under and overestimation of the computed freeboard. Therefore, we implemented a limiting scheme for the interpolated sea surface height to keep the converted freeboard values within physically reasonable limits. We derived the lower level of the sea surface height from the knowledge that all elevations between two open water points represent ice. Thus, the sea surface height in this region must be lower than the measured elevations. We determined the lower envelope of surface for each point measurement as the minimum elevation in 500 by 500 laser point measurements, which corresponds at a typical flight height of 1000 ft to a spatial scale of 160 m. Wherever the interpolated sea surface height was higher than the lower envelope of the sea-ice surface, we set the sea surface height to the lower envelope plus a mimimum freeboard of 0.05 m, which is the assumed elevation uncertainty. In doing so, the minima spanning the lower envelope will have a freeboard of 0.05 m in the later conversion. We also limited the sea surface height for strong positive undulations in the elevation between open water points that would result in erroneously high freeboard estimates. We capped the sea surface height such that the lower envelope of the surface did not exceed it by more than 2 m. We did not apply either of the limits 20 m around open water points but subsequently ramped them to be fully active 50 m away from open water points. Similar to the spatial ramping, we also ramped both criteria over a freeboard range of 0.1 m to prevent a sharp leveling off the sea surface height. We stored all corrections to the interpolated sea level height in the uncertainty term *σ*_*fb*_*limit*_. To ensure consistent application of the limiting procedure to both transect and floe grid flights, we applied it to each 30-second segment individually. This approach can introduce small discontinuities in the sea surface height between segments (Figs. 11 and 12), which we accounted for in the freeboard uncertainty. In future data releases, this issue could be addressed for floe grid flights by performing the limiting of the sea surface height for the entire floe grid instead of 30-s segments.

#### Elevation offset correction

In this section, we outline a method to use regions of overlapping flight tracks from the floe grid surveys to quantify elevation offsets and to correct for them without losing spatial details of the sea-ice surface. The principle idea of this correction is that the elevation signal measured by the ALS, ${h}^{{\prime} }\left(x,y,t\right)$ at time *t*, consists of two parts, the true elevation $h\left(x,y\right)$ and the offset in the undulations in the elevation $c\left(t\right)$,

3 $${h}^{{\prime} }\left(x,y,t\right)=h\left(x,y\right)+c\left(t\right).$$

For an overlapping region, the following relationship holds,

4 $${h}^{{\prime} }\left(x,y,{t}_{i}\right)+c\left({t}_{i}\right)={h}^{{\prime} }\left(x,y,{t}_{j}\right)+c\left({t}_{j}\right)\,\;\iff \;\,c\left({t}_{j}\right)-c\left({t}_{i}\right)={h}^{{\prime} }\left(x,y,{t}_{i}\right)-{h}^{{\prime} }\left(x,y,{t}_{j}\right),$$

where *t*_*i*_ and *t*_*j*_ are the times where the ALS mapped the surface at the same position $\left(x,y\right)$. We could determine the elevation offset from this relationship in case of frequent overlapping regions by solving for the $c\left(t\right)$ that minimized all differences of the measured elevation in overlapping regions. We binned all timestamps of overlapping regions into the 500 discrete time bins equally spaced over the flight duration to formulate Eq. (4) as a minimization problem in matrix form,

5 $$\mathop{\underbrace{\left[\begin{array}{cccccccccc}0 & \ldots  & 0 & -1 & 0 & \ldots  & 0 & 1 & 0 & \ldots \\  &  &  &  &  & \vdots  &  &  &  & \end{array}\right]}}\limits_{{\bf{M}}}\mathop{\underbrace{\left[\begin{array}{c}c({t}_{0})\\ \vdots \\ c({t}_{i})\\ \vdots \\ c({t}_{j})\\ \vdots \\ c({t}_{n})\end{array}\right]}}\limits_{{\bf{c}}}=\mathop{\underbrace{\left[\begin{array}{c}{h}^{{\rm{{\prime} }}}(x,y,{t}_{i})-{h}^{{\rm{{\prime} }}}(x,y,{t}_{j})\\ \vdots \end{array}\right]}}\limits_{{\bf{h}}}.$$

The number of overlapping grid cells determined the number of rows of the matrix **M**. In the initialisation of **M** and **h**, we took only every 100th overlapping grid cell into account when solving for $c\left(t\right)$ to balance numerical costs and the high information redundancy due to the very high spatial resolution of the ALS data. Finally, we determined $c\left(t\right)$ by a least-square fit of Eq. (5). In the following, we outline two modifications to Eq. (5) to account for the known elevation of open water points and to dampen strong variations of the elevation in regions with few open water points.

Open water (ow) points are the only locations of the surveyed area for which the true ellipsoidal elevation is known and given by sea level height $ssh\left(x,y\right)$. Thus the correction term at these tie points is given by,

6 $$c\left({t}_{ow}\right)={h}^{{\prime} }\left({x}_{ow},{y}_{ow},{t}_{ow}\right)-ssh\left({x}_{ow},{y}_{ow}\right).$$

We appended matrix **M** by one row for each open water point that was filled with zeros for all entries except the column corresponding to the timestamp of the open water point where the matrix entry was set to 1. Accordingly, we appended vector **h** by the known correction term in Eq. (6) for all open water points. All entries in columns of the matrix **M** corresponding to temporal bins of open water points $c\left({t}_{ow}\right)$ were set to 0. In the solution vector **h**, we subtracted the known correction term (Eq. (6))  c(*t*_*ow*_ = *t*_2_) or added c(*t*_*ow*_ = *t*_1_) for all rows of the matrix **M** that were modified. With these modifications to **M** and **h**, the correction term for open water points was fixed, while the correction term for all other temporal bins was determined by the least square fit.

The outlined offset correction will correct for the relative difference between overlapping elevation measurements. However, the absolute value of the correction term is only constrained by open water points. For winter flights with strong elevation undulations, too few open water points can lead to large scale variations in the corrected terms that are unrealistic. Therefore, we introduced tendencies in the correction term computation for all flights with poor RTNav GNSS altitude quality. These tendencies pulled all corrected elevations towards the expected ice elevation. We determined the expected ice elevation by interpolating the elevation of the ice surrounding detected open water points, computed as the mean elevation in a radius of 100 m around those points, to all times of the flights. Given the proximity to open water with known ellipsoidal elevation, the elevations in these areas are more reliable than elevations farther away from open water. We added these tendencies as new rows to the matrix **M** and the solution vector **h** similarly as the fixed correction terms for the open water points, but the remaining parts of **M** and **h** were not changed, such that the tendencies were just additional constraints in the computation of $c\left(t\right)$.

**Fig. 5:**
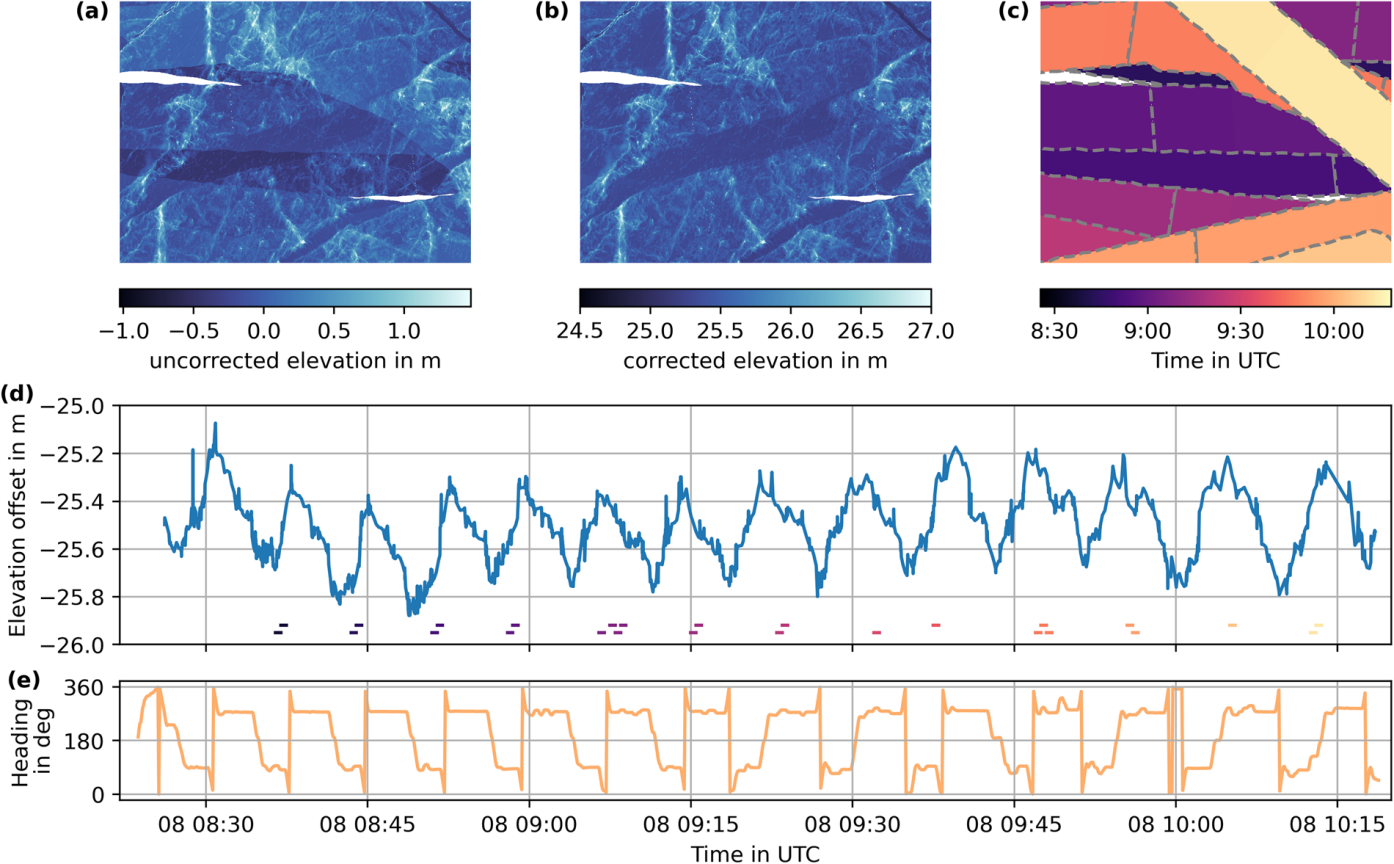
(**a**) Snow/ice surface elevation in a 1.5 × 1.0 km^2^ subdomain of the April 8th flight before elevation offset correction. Uncorrected swaths from different overflights can be seen. (**b**) Ellipsoidal snow/ice surface elevation for the same subdomain after elevation offset correction is applied, providing consistent elevations for different overflights and referencing open water points to the mean sea surface height (DTU21). (**c**) Timestamps of the individual elevation point measurements in the domain. 30-s segments are surrounded by gray dashed lines. (**d**) The computed elevation offset is a function of flight time. The timing of all 30-s segments covering the subregion is given in bars at the bottom of the plot using the same colormap as (**c**). (**e**) True heading of aircraft during the survey.

Once we constructed both matrix **M** and the solution vector **h** from the overlapping regions, the open water points, and the tendencies, we solved the linear system in Eq. (5) by a least-square fit. Figure 5d shows the computed elevation correction term for the April 8, 2020 flight. The undulations are sensitive to the flight path of the survey most likely linked to shadowing of GNSS signals by the helicopter’s frame depending on flight direction (Fig. 5e). We provide a more sophisticated analysis of the computed elevation offsets, the associated spatial and temporal scales of the undulations, and their impact on elevation and freeboard quality in the section Technical Validation. Figure 5a,b illustrates how correcting the measured elevation with the computed offset correction term leads to consistent elevations for different overflights of the survey area.

We quantified the effect of the elevation correction on the uncertainty of the derived freeboard values *σ*_*fb*_*hcor*_ as the differences of the correction terms at each point compared to their closest open water points. The overall uncertainty of the freeboard data was then given by the sum of contributions from the elevation correction and the sea surface height limiting *σ*_*fb*_ = *σ*_*fb*_*hcor*_ + *σ*_*fb*_*limit*_.

#### Processing steps for different survey types

In this section, we outline the different processing steps for different survey types. In general, we distinguished two different survey types: (1) floe grids, where the helicopter flew a pattern of multiple parallel and overlapping lines over the MOSAiC CO to map the sea-ice surface in an approximately 5 × 5 km^2^ area; and (2) transects with little to no swath-to-swath overlap of the surveyed area. The main differences in the processing between the survey types were the amount of overlapping survey area that allowed to correct for undulations in the elevation, and the proximity to Polarstern that allowed for the correction of ice drift during the flight.

We processed the floe grid surveys in two iterations (Fig. 2b). First, we applied an atmospheric backscatter filter to the ALS point cloud data and performed an ice drift correction. We gridded all data and merged them into one floe grid to compute and store the correction term for the undulations in the elevations. In the second iteration, we ran the open water detection on the data corrected for the elevation offset and ice drift and then performed the freeboard conversion. We then gridded all parameters in 30-s segments and merged all segments into one floe grid.

For transects and all other survey flights, the processing included the following steps (Fig. 2c): atmospheric backscatter filter, open water detection, freeboard conversion, and gridding of all parameters in 30-s segments. We did not perform corrections for elevation offset nor for sea-ice drift.

## Data Records

In total, this data set includes 35 floe grid and 29 transect surveys between September 2019 to September 2020 along the drift path of the MOSAiC expedition (Fig. 1). We list information of all surveys including date, leg, position, and GNSS quality in Table 4 for floe grid surveys and in Table 5 for transect flights. For each flight, we provide data at different processing levels together with auxiliary information as specified in the sections below. Raw sensor data and data products of intermediate processing steps are planned to be released at a later point in time. We summarize the data variables contained in each data product, depending on the GNSS quality, in Table 6. The presented data are available at PANGAEA, please see the sections below for references to the specific datasets.

**Table 4:** Details on all 35 floe grid flights that include swath-to-swath correction of elevation.

Date	Flight ID	Device operation	Data start	Data end	Leg	Position	GNSS quality
2019 / 10 / 02	20191002_01	PS122/1_2-57	06:05	06:44	1	134.46^°^E 85.17^°^N	PPP
2019 / 10 / 20	20191020_01	PS122/1_2-167	01:07	02:07	1	132.80^°^E 84.97^°^N	PPP
2019 / 11 / 12	20191112_02	PS122/1_7-25	10:28	11:00	1	117.53^°^E 86.03^°^N	RTNav
2019 / 11 / 19	20191119_01	PS122/1_8-23	04:02	04:48	1	120.46^°^E 85.80^°^N	RTNav
2019 / 11 / 30	20191130_01	PS122/1_9-98	04:50	05:30	1	113.07^°^E 86.06^°^N	RTNav
2019 / 12 / 24	20191224_01	PS122/2_17-98	07:40	09:16	2	113.79^°^E 86.63^°^N	RTNav
2019 / 12 / 25	20191225_01	PS122/2_17-99	14:07	15:40	2	115.11^°^E 86.63^°^N	RTNav
2019 / 12 / 28	20191228_01	PS122/2_17-101	07:06	08:15	2	115.76^°^E 86.68^°^N	RTNav
2020 / 01 / 07	20200107_01	PS122/2_19-44	09:22	10:52	2	114.95^°^E 87.12^°^N	RTNav
2020 / 01 / 08	20200108_01	PS122/2_19-46	07:54	08:34	2	115.22^°^E 87.11^°^N	RTNav
2020 / 01 / 08	20200108_03	PS122/2_19-52	09:43	10:23	2	115.21^°^E 87.11^°^N	RTNav
2020 / 01 / 08	20200108_04	PS122/2_19-53	12:49	13:32	2	115.16^°^E 87.11^°^N	RTNav
2020 / 01 / 16	20200116_01	PS122/2_20-52	06:34	07:57	2	101.95^°^E 87.56^°^N	RTNav
2020 / 01 / 21	20200121_01	PS122/2_21-41	10:35	12:28	2	96.13^°^E 87.49^°^N	RTNav
2020 / 01 / 23	20200123_02	PS122/2_21-78	13:23	14:16	2	93.88^°^E 87.44^°^N	RTNav
2020 / 01 / 28	20200128_01	PS122/2_22-16	06:34	08:07	2	95.82^°^E 87.45^°^N	RTNav
2020 / 02 / 04	20200204_01	PS122/2_23-14	07:15	09:10	2	95.22^°^E 87.47^°^N	RTNav
2020 / 02 / 12	20200212_01	PS122/2_24-31	08:15	10:31	2	87.73^°^E 87.87^°^N	RTNav
2020 / 02 / 17	20200217_02	PS122/2_25-8	09:00	10:49	2	79.01^°^E 88.07^°^N	RTNav
2020 / 02 / 27	20200227_01	PS122/3_29-49	11:50	12:53	3	36.64^°^E 88.41^°^N	RTNav
2020 / 03 / 18	20200318_01	PS122/3_32-42	10:51	10:56	3	12.14^°^E 86.69^°^N	RTNav
2020 / 03 / 21	20200321_01	PS122/3_32-70	08:13	10:08	3	15.19^°^E 86.26^°^N	RTNav
2020 / 04 / 08	20200408_01	PS122/3_35-49	08:25	10:18	3	14.65^°^E 84.48^°^N	PPP
2020 / 04 / 23	20200423_01	PS122/3_37-63	07:44	09:31	3	16.05^°^E 84.08^°^N	PPP
2020 / 05 / 10	20200510_01	PS122/3_39-109	13:58	15:43	3	13.39^°^E 83.64^°^N	PPP
2020 / 06 / 16	20200616_01	PS122/4_44-78	09:22	10:46	4	8.32^°^E 82.21^°^N	PPP
2020 / 06 / 30	20200630_01	PS122/4_45-36	07:40	09:08	4	8.98^°^E 81.80^°^N	PPP
2020 / 07 / 04	20200704_01	PS122/4_45-112	09:11	10:31	4	6.80^°^E 81.71^°^N	PPP
2020 / 07 / 07	20200707_01	PS122/4_46-36	08:58	10:08	4	4.66^°^E 81.65^°^N	PPP
2020 / 07 / 17	20200717_01	PS122/4_47-96	15:22	17:17	4	0.36^°^E 81.13^°^N	PPP
2020 / 07 / 22	20200722_01	PS122/4_48-69	15:15	17:28	4	-0.56^°^E 80.46^°^N	PPP
2020 / 09 / 07	20200907_01	PS122/5_61-62	04:22	06:16	5	112.07^°^E 88.72^°^N	RTNav
2020 / 09 / 11	20200911_01	PS122/5_61-190	05:33	07:41	5	104.57^°^E 88.74^°^N	RTNav
2020 / 09 / 15	20200915_01	PS122/5_62-67	04:45	06:20	5	107.65^°^E 89.07^°^N	PPP
2020 / 09 / 19	20200919_01	PS122/5_62-166	10:04	12:02	5	110.46^°^E 89.14^°^N	RTNav

Data start and end times are given in UTC. PPP stands for precise point positioning and RTNav for real-time navigation solutions.

**Table 5:** Details on all 29 flights processed as transects without swath-to-swath elevation correction, which include L-site triangle surveys, event surveys or floe grid flights with limited coverage.

Date	Flight ID	Device operation	Data start	Data end	Leg	Position	GNSS quality
2019 / 09 / 28	20190928_01^*^	PS122/1_2-45	04:14	04:28	1	119.81^°^E 82.18^°^N	PPP
2019 / 10 / 29	20191029_01	PS122/1_5-9	02:05	03:20	1	125.59^°^E 85.64^°^N	RTNav
2019 / 11 / 05	20191105_01^*^	PS122/1_6-11	06:31	07:26	1	118.72^°^E 85.95^°^N	RTNav
2019 / 11 / 12	20191112_01	PS122/1_7-24	08:01	09:07	1	117.50^°^E 86.02^°^N	RTNav
2019 / 12 / 06	20191206_01	PS122/1_10-78	06:58	08:13	1	122.26^°^E 86.14^°^N	RTNav
2019 / 12 / 30	20191230_01	PS122/2_18-7	08:02	09:34	2	116.68^°^E 86.60^°^N	RTNav
2020 / 01 / 07	20200107_02	PS122/2_19-45	12:19	13:49	2	114.98^°^E 87.12^°^N	RTNav
2020 / 01 / 08	20200108_02^*^	PS122/2_19-51	08:54	08:55	2	115.22^°^E 87.11^°^N	RTNav
2020 / 01 / 16	20200116_02	PS122/2_20-53	09:19	10:49	2	101.70^°^E 87.56^°^N	RTNav
2020 / 01 / 23	20200123_01	PS122/2_21-77	10:20	12:13	2	93.96^°^E 87.44^°^N	RTNav
2020 / 01 / 25	20200125_01^*^	PS122/2_21-122	12:45	14:21	2	92.84^°^E 87.40^°^N	RTNav
2020 / 02 / 02	20200202_01	PS122/2_22-97	06:43	07:04	2	95.41^°^E 87.34^°^N	RTNav
2020 / 02 / 09	20200209_01	PS122/2_23-109	11:05	12:37	2	92.93^°^E 87.73^°^N	RTNav
2020 / 02 / 17	20200217_01	PS122/2_25-7	06:52	08:19	2	79.17^°^E 88.07^°^N	RTNav
2020 / 03 / 21	20200321_02	PS122/3_32-71	10:27	11:51	3	15.23^°^E 86.26^°^N	RTNav
2020 / 03 / 23	20200323_01^*^	PS122/3_33-17	10:39	12:16	3	15.81^°^E 86.21^°^N	RTNav
2020 / 04 / 07	20200407_01	PS122/3_35-48	07:53	09:34	3	14.53^°^E 84.50^°^N	PPP
2020 / 04 / 23	20200423_02	PS122/3_37-66	10:07	11:26	3	16.03^°^E 84.07^°^N	PPP
2020 / 06 / 11	20200611_01^*^	PS122/4_44-27	12:56	13:06	4	8.95^°^E 81.67^°^N	PPP
2020 / 06 / 15	20200615_02^*^	PS122/4_44-65	12:16	13:28	4	8.20^°^E 82.05^°^N	PPP
2020 / 06 / 30	20200630_02	PS122/4_45-37	09:35	11:01	4	8.98^°^E 81.80^°^N	RTNav
2020 / 07 / 07	20200707_03	PS122/4_46-39	12:28	14:27	4	4.41^°^E 81.63^°^N	PPP
2020 / 07 / 11	20200711_01^*^	PS122/4_46-97	11:21	12:08	4	1.55^°^E 81.45^°^N	PPP
2020 / 08 / 06	20200806_01^*^	PS122/4_50-32	08:54	10:42	4	-7.24^°^E 78.35^°^N	PPP
2020 / 08 / 07	20200807_01^*^	PS122/4_50-45	08:35	10:17	4	-5.11^°^E 79.68^°^N	PPP
2020 / 08 / 18	20200818_02^*^	PS122/5_59-139	15:39	16:20	5	-35.66^°^E 89.10^°^N	RTNav
2020 / 09 / 08	20200908_02^*^	PS122/5_61-63	12:23	13:51	5	112.96^°^E 88.68^°^N	RTNav
2020 / 09 / 21	20200921_01^*^	PS122/5_63-3	07:46	09:37	5	106.19^°^E 88.51^°^N	RTNav
2020 / 09 / 28	20200928_02^*^	PS122/5_63-118	11:11	12:15	5	25.20^°^E 83.15^°^N	PPP

Data start and end times are given in UTC. PPP stands for precise point positioning and RTNav for real-time navigation solutions.

^*^ indicated event flights with varying spatial coverage. All other flights are L-site triangle flights covering the same region.

**Table 6:** Data variables are provided for different flight types and GNSS accuracy.

Flight type	GNSS	Helicopter GNSS/INS	Point cloud data	Gridded 30-s seg-ments	Merged floe grids
**floe grid**	**PPP**	time distance easting northing ellipsoid height latitude longitude ellipsoid height roll pitch heading east velocity north velocity up velocity east sd north sd height sd roll sd pitch sd heading sd	elevation elevation reference reflectance echo width	elevation (corrected) elevation reference freeboard freeboard uncertainty reflectance echo width sea surface height	elevation (corrected) elevation reference freeboard freeboard uncertainty reflectance echo width sea surface height
	**RTNav**			elevation (corrected) elevation reference freeboard estimate freeboard uncertainty reflectance echo width sea surface height	elevation (corrected) elevation reference freeboard estimate freeboard uncertainty reflectance echo width sea surface height
**transect**	**PPP**			elevation elevation reference freeboard freeboard uncertainty reflectance echo width sea surface height	—
	**RTNav**			elevation elevation reference reflectance echo width	—

PPP stands for precise point positioning, RTNav for real-time navigation solutions, and sd for standard deviation.

### GNSS/INS data

The GNSS/INS data^29^ contain a combined record of the helicopter’s position and attitude data either at 10 Hz (RTNav) or 200 Hz (PPP) as plain text, fixed-width tables with a header (*.txt). The file sizes depend strongly on the GNSS solution and on the length of the flights but are typically 1–20 MB (RTNav) or 100–300 MB (PPP).

### Elevation point clouds

The elevation point clouds^30^ consist of sea-ice surface elevation data retrieved for each laser shot (as outlined in sections Preprocessing and Elevation retrieval as well as in Fig. 2a). The data are stored in arrays with the dimensions representing the number of scan lines (along-track, scan rate) and the number of shots per scan line (across-track, field-of-view resolution) that depend on the measurement program used (Table 2). The regular angular and temporal spacing of the original laser data result in an irregular spatial spacing due to the changing speed, altitude, position, and attitude of the helicopter during the flight. The data are provided in custom binary format (*.alsbin2). One file covers nominally up to 5 min of flight time and has a size of 2–3 GB depending on the measurement program. Therefore, a full 2-h survey in point cloud data can reach a total size of up to 72 GB. Each file is accompanied by a metadata file (*.json) and a quicklook plot (*.png) archived together as *.zip.

### Gridded 30-s segments

The gridded 30-s segments^31^ contain data that are further processed from the point cloud data by applying various filters and corrections depending on the survey type (see section Processing of regular-gridded data and Fig. 2b,c) and by linearly interpolating the data onto a regular spatial grid with a resolution of 0.5 m. The data together with metadata are provided in the format of Network Common Data Form version 4 (netCDF4, *.nc). One file covers 30 s of flight time and has a typical size of 1–150 MB depending on the measurement program and on the helicopter’s altitude, position, and attitude. Therefore, a full 2-h survey as 30-s segments can reach a total size of 36 GB. Data of each flight are accompanied by a list of detected open water points (*.csv) and an overview plot (*.png).

### Merged floe grids

The merged floe grids^32^ combine the individual 30-s segments of the local-scale floe grid flights into one map. For each flight, the data are provided as a single netCDF4 file (*.nc) with metadata or alternatively as individual variables in GeoTIFF format (*.tiff). The size of a merged floe grid netCDF file depends on the survey extent and varies at 250 MB–8 GB while individual GeoTIFFs have smaller sizes of 70 MB–4 GB. Data from each flight are accompanied by the computed elevation correction term (*.csv).

## Technical Validation

There are four major sources of uncertainty in the presented surface elevation and freeboard data that could degrade the data quality, originating from (1) the precision of the GNSS/INS position and attitude of the helicopter; (2) the uncertainty of the laser scanner and the classification of laser returns (atmospheric backscatter or sea-ice/snow surface); (3) the sea-ice conditions at the time of survey (ice drift and presence of leads); and (4) the uncertainty of the interpolated sea surface height. The largest contribution to the uncertainty arises from the combination of poor GNSS quality and few open-water points in a closed winter ice-pack, while the accuracy and the precision of the laser scanner are high (both 25 mm)^14^, the atmospheric backscatter is filtered reliably by limiting the allowed range of elevations, and Polarstern’s position allows for a reasonable correction of the ice drift during the survey.

### Degraded INS/GNSS solutions

**Fig. 6:**
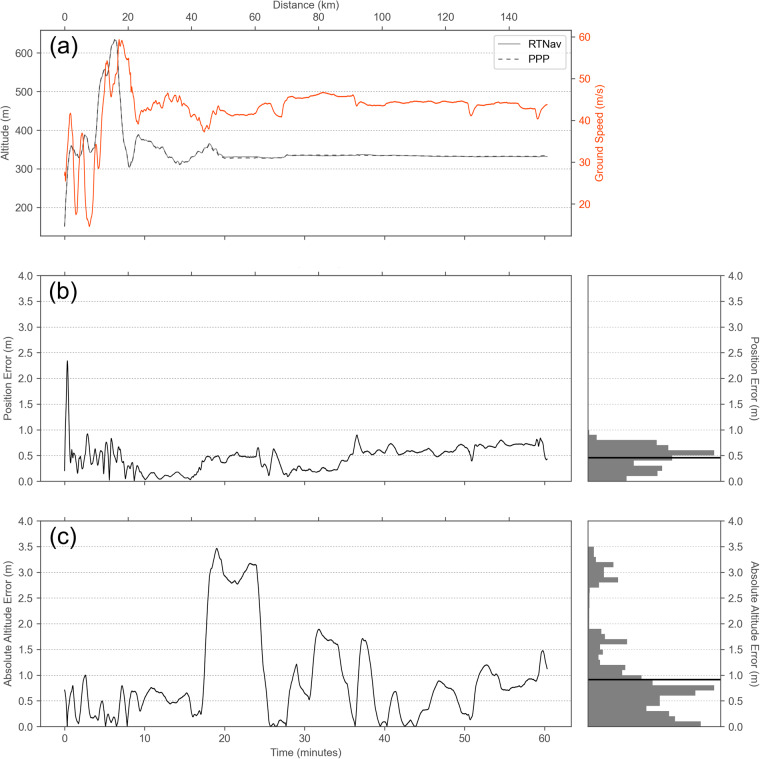
Altitude and positional error computed from differences in the precise point positioning (PPP) and real-time navigation (RTNav) solutions from a survey on June 30, 2020, assuming PPP is the true position. (**a**) Survey altitude and ground speed. (**b**) Positional error from the horizontal offset at ground level with a histogram of error values. (**c**) Altitude error from the vertical offset with a histogram of error values.

To test the level of degradation of RTNav with respect to the PPP solution, we compared the two solutions for one survey on June 30, 2020. Here, the PPP solution for longitude, latitude, and altitude was re-sampled to the 10 Hz RTNav solution. We took the PPP solution as truth and computed the altitude error and positional error as vertical and horizontal deviations between the two solutions for approximately one flight hour with a track length of 150 km (Fig. 6).

The mean position error is 0.46 m and the mean altitude error is 0.91 m, but outliers exist with values well above 2 m for both parameters. Both errors contain high-frequency variations, which are often seemingly unrelated to changes in flight altitude. Altitude error is directly related to elevation error and this magnitude is an unacceptable uncertainty for mapping sea-ice surface height. But there is the possibility to mitigate the altitude error by using open water or thin ice in leads along the flight track as reference elevation. Such reference elevation tie points must be available at sufficient frequency to characterize and correct the high-frequency variations of the altitude error. This is a scenario for a longer transect survey, without overlaps of adjacent swaths that could also be used to mitigate the altitude error.

**Fig. 7:**
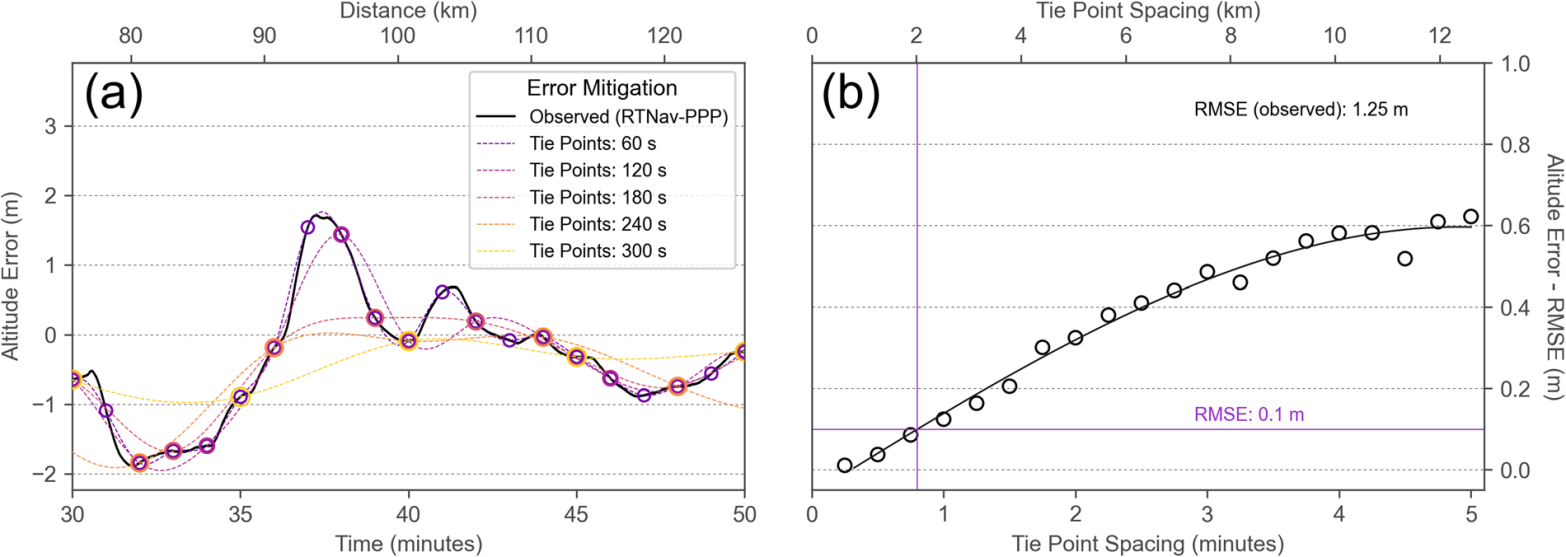
Altitude error mitigation strategy assuming the altitude error can be obtained at regular intervals. (**a**) Example of error mitigation strategy. Altitude error is estimated at regular intervals (shown only from 1 to 5 minutes in 1-minute steps). With an average ground speed of 42 m s^−1^, the covered distance within 1 minute is roughly 2.5 km. This panel is a subset of Fig. 6c. (**b**) Root-mean-square-error (RMSE) that can be obtained from altitude error correction tie points with different frequencies. An RMSE value of 0.1 m requires a reference tie point spacing of roughly 48 seconds or 2 km. The observed RMSE (without correction) is 1.25 m.

To determine the frequency of leads that would be needed to reduce the altitude error to a certain level, we sampled the observed altitude error in intervals of multiples of 15 seconds, computed a spline interpolation between these tie points, and applied the correction to the RTNav altitude. We then computed the difference between the corrected RTNav altitude and the PPP reference altitude. The metric for the comparison is the root mean square error (RMSE) as a measure for uncertainty (Fig. 7). Other error components, such as attitude error will also contribute to the elevation error budget, but these are assumed to be negligible.

As expected, the RMSE of the altitude error can be reduced considerably if the correction terms are available at intervals below 1 minute for the entire survey. 1 minute of flight time roughly corresponds to 2.5 km with an average ground speed of 42 m s^−1^ of this particular flight. If we consider a target value of 10 cm for elevation accuracy, a correction tie point every 48 seconds or 2 km is needed. Unfortunately, this lead frequency is not realistic for the winter conditions in the central Arctic Basin. For example, during the CO floe grid flight at 88. 4° N on February 27, 2020, mere 12 open water points were detected along the approximately 200 km long flight track. It should be noted, however, that even if a reference elevation can only be found every 5 minutes, this still results in a better RMSE value of roughly 0.6 m, compared to the 1.25 m of the observed altitude error without any corrections.

We therefore conclude that the RTNav horizontal positioning and attitude information may still be adequate for the purpose of the processing of the ALS data, although the vertical position uncertainty is insufficient for surveys with only the RTNav solution available. We also have observed that the flight altitude for RTNav solutions shows large undulations up to 10 m over spatial scales larger than 10 km. We hypothesize that this issue is linked to shadowing of the GPS/GNSS antennae by the aircraft frame depending on flight direction (see section Elevation offset correction). In the PPP solutions, such an effect would be mitigated by the merging with accelerations measured by the INS and the magnitude in PPP flights is rather 0.5 m (see below the section on Effect of elevation correction).

### Application of uncorrected elevation: surface roughness

**Fig. 8:**
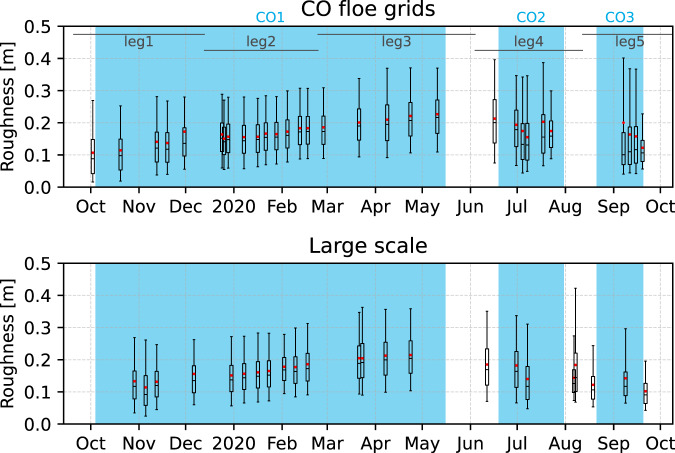
Evolution of surface roughness distribution (inferred from the standard deviation of detrended scan line elevation) over the CO floe grids (top) and large scale surveys (bottom). The whiskers show the interdecile range (10th to 90th percentiles), boxes show the interquartile range (25th to 75th percentiles), black lines are the medians, and red dots are the means of inferred surface roughness values. Outliers are not shown. The duration of the different legs of MOSAiC are shown at the top and the different Central Observatories (CO) are marked in blue according to Nicolaus *et al*.^13^.

Even in the case of poor GNSS altitude solution, the ALS surface elevation data can still provide valuable insight into the sea-ice surface as small-scale features are well captured and rather unaffected by the elevation offset (see below the section Effect of elevation correction). Following the approach of Beckers *et al*.^33^, we can calculate estimates for sea-ice surface roughness from the ALS data. In the point cloud data, each scan line contains up to about 1000–1500 elevation values covering a swath width of about 60–300 m depending on the measurement program and flight altitude. First, we removed any atmospheric backscatter by applying the same filter as in processing the gridded data. Then, we detrended the scan line elevations to remove any possible cross-track drift in elevation due to the poor GNSS altitude solution. Finally, we took surface roughness as the standard deviation of surface elevation along each scan line. In Fig. 8, the box-and-whisker plots show the distributions of surface roughness for each CO floe grid and large-scale transect flight. The data show a general increase of surface roughness due to large-scale deformation (pressure ridges) in addition to snow accumulation and redistribution (snow dunes) until the end of winter in April/May with roughness values typical to Arctic sea ice and comparable to other studies^33,34^. Later on, there is a general decrease in surface roughness indicating melt, although we acknowledge that the surveyed area was different and areas of open water were not filtered out causing loss of data. We also note that, in this demonstrative example, the data contain the entire duration of the flights regardless of their length as well as exact spatial extent and overlap, which were not matched with each other.

### Effect of elevation correction

**Fig. 9:**
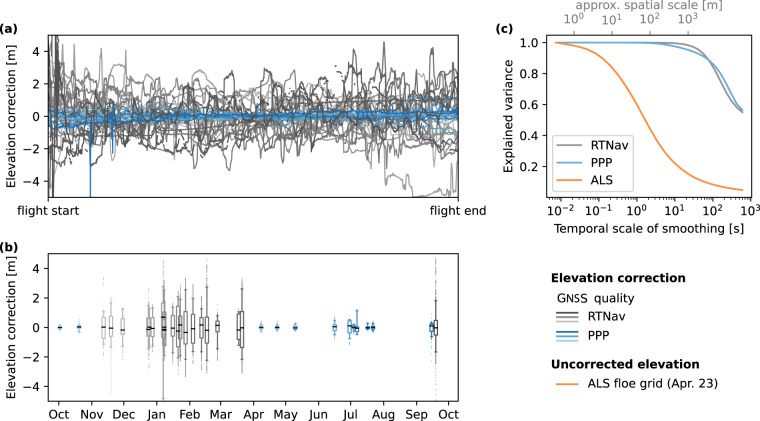
Details on the computed elevation offset that document the impact of the GNSS quality: the temporal evolution of the elevation offset during each flight is given in (**a**). To highlight the temporal variability, we subtracted the mean elevation offset for each flight. The seasonal evolution of the elevation offset is given in one boxplot per flight in (**b**). The box indicates the interquartile range (25th to 75th percentile), the whiskers the 5th and 95th percentile, the median is given as a black line, and outliers as dots. To determine the spatial and temporal scales of variability in the elevation offset, we low-pass filtered the elevation offsets over different scales and compute the amount of explained variance at different spatial and temporal scales in (**c**). As a reference of the scales dominating the variability of the surface elevation, we performed the same analysis for the center beam elevation of the ALS floe grid survey from April 23rd 2020. We estimated the spatial scales denoted in (**c**) from the temporal scales assuming constant speed of the helicopter (Table 2). PPP stands for precise point positioning and RTNav for real-time navigation solutions.

For floe grid surveys, we used overlapping helicopter passes to estimate the uncertainty related to vertical GNSS positioning. The resulting time-dependent offset in elevation is shown in Fig. 9a. In general, flights with only RTNav positioning show much higher elevation offsets up to 10 m compared to surveys with precise point positioning. As the availability of the PPP depends on the latitude of the flights, the wintertime flights from November till April show the largest offsets as well as leg 5 flights close to the North Pole (Figs. 1a and 9b). The offset does not follow one characteristic shape for all flights but is rather linked to the pattern of the flight track of the helicopter as shown in Fig. 5d,e, which is most likely related to shadowing of the GPS/GNSS antennae by the aircraft. To better understand what impact the elevation undulations potentially have on sea-ice characteristics that we aim to deduce from the ALS data, we examined closer their dominant spatial and temporal scales of variability. The idea here is that if the undulations vary at much larger scale than surface elevations themselves, we are still able to study small-scale surface features, as for example roughness as shown above. To determine the different scales of variability, we low-pass filtered the elevation offset over different temporal scales by smoothing the time series with a sliding window of varying size. Next, we determined how much of the total variance of the elevation offset time series can be explained by the variance of the filtered time series. If at a certain time scale the value of 1 is reached, all variance takes place at higher temporal scales. For a value of 0, all variability is explained by variations at smaller temporal scales. We performed this analysis for all floe grid surveys and the results grouped and averaged by RTNav and PPP are shown in Fig. 9c. We also applied this method to the uncorrected surface elevation of the center beam elevation during the floe grid survey from April 23rd 2020, to get a reference for the temporal scales that dominate the variability of the sea-ice surface. This was the flight with the lowest elevation offset where we expect that the determined scales of variability were characteristic of the sea-ice surface and not a signal of the offset. Finally, we converted the temporal scales of variability to spatial scales using the average speed of the helicopter (Table 2). In the elevation data, the explained variance quickly decreases with increasing temporal scale (Fig. 9c) and around 60 % of the total variance is explained by small-scale features ( < 100 m), e.g. pressure ridges. We found negligible low variability in the elevation offset below 100 s or 5000 m (explained variances are close to 1). This means that variations in the surface elevation at scales larger than 100 s or 5000 m are likely impacted by the elevation undulations. However, surface elevation variations at scales larger than 5000 m only account for 8 % of the total variance in the surface elevation. This highlights both the potentials and limitations of the presented elevation data especially for flights with RTNav positioning: small-scale features are well documented in the presented data as shown in our analysis of surface roughness above (Fig. 8), whereas large-scale surface variability such as transitions from thinner to thicker ice regimes might be distorted by the elevation offset. The conversion from surface elevation to freeboard reduces if not eliminates these large-scale variations, but only if leads are present frequently during the flight (as discussed above and shown in Fig. 7). Unfortunately, the flights with the highest elevation offset are during wintertime when there was a very limited number of open water points.

### Comparison with ICESat-2 freeboard product

**Fig. 10:**
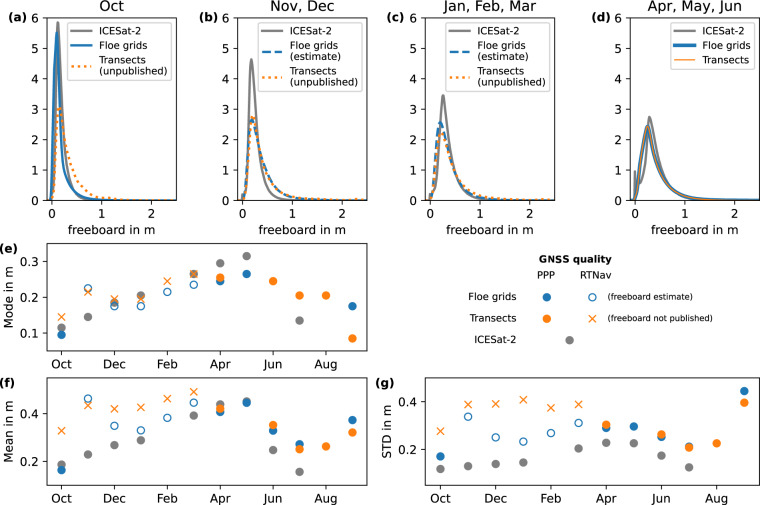
Comparison of freeboard distributions from ALS and ICESat-2 data acquired within 100 km around Polarstern. (**a**) to (**d**) show freeboard distributions averaged over different month groups for floe grid (blue) and transect flights (orange). The seasonal evolution of the distributions is shown as a time series of the mode (**e**), the mean (**f**), and the standard deviation (**g**). Different line styles and markers denote GNSS quality during the respective months for the different survey types. PPP stands for precise point positioning and RTNav for real-time navigation solutions.

To asses our ALS freeboard product, especially for RTNav flights, over larger scales and a seasonal cycle, we compared them with data from the Ice, Cloud and land Elevation Satellite-2 (ICESat-2) available in the larger region around the MOSAiC site. We sampled all ICESat-2 ATL10 freeboard data^35^ (weak and strong beams) within 100 km around Polarstern and computed monthly freeboard distributions. We chose this large region to correspond to the regional surveys and to account for the sparse sampling of ICESat-2 due to clouds, limited open water points, and polar coverage gap > 88° N. We compared the monthly freeboard distributions from ICESat-2 to the monthly freeboard distribution of the ALS flights grouped by survey type (floe grid and transects) as well as GNSS quality (Fig. 10). We compared the distributions for different groups of months (Fig. 10a–d), as well as the seasonal cycle of the mode, mean, and standard deviation of the monthly distributions (Fig. 10e–g). For ICESat-2, we observe a steady shift of the mode (Fig. 10e) associated with a broadening of the freeboard distribution (Fig. 10g) from the freeze-up to melt season. For the ALS data, we find distributions that closely match the ICESat-2 data for the periods where PPP GNSS quality is available (October and from April onwards, Fig. 10a,d). In November, however, we observe an instant increase in the standard deviation by 0.17 m (floe grids) and 0.11 m (transects) associated with a broadening of the distribution (Fig. 10g). The standard deviation remains nearly constant over the entire period with RTNav GNSS quality for transect flights. For floe grids, we observe this instant broadening only in November, while in the other months with RTNav GNSS quality the difference in standard deviation between ALS and ICESat-2 is in a range comparable to the months with PPP data quality. For the entire RTNav period (except for November), the modes of the distributions match well the ICESat-2 distributions for both survey types (Fig. 10f). In general, the ALS distributions are expected to be broader given the higher spatial resolution of the freeboard data (0.5 m compared to about 15–30 m for ICESat-2 ATL10 segments) as shown by a direct comparison in Ricker *et al*.^36^ because the ALS data resolve more ridges. This broadening is visible as the moderate difference in the standard deviation between ALS and ICESat-2 for the PPP flights. The higher standard deviations for months with RTNav flights, however, are linked to the combination of strong elevation offsets and the limited amount of open water during that time. We find that correcting for the elevation offset in floe grid flights significantly reduces this broadening of the distributions, leading to standard deviations in a range comparable to months with PPP GNSS quality (with the exception of November). However, for RTNav transect flights, where a swath-to-swath correction was not possible due to the limited overlap, we refrain from providing freeboard data products given that the differences between ALS and ICESat-2 freeboard distributions with respect to mean and standard deviations are large. The user can produce freeboard estimates of the quality documented in Fig. 10 for these flights using the elevation data products and the detected open water points, both of which are provided (see Section Data Records).

## Usage Notes

### GNSS/INS data

The helicopter’s position and attitude data are provided as plain text tables and do not require specific software to be read. The main use case of these data is the georeferencing of the data recorded by various instruments during the flight, which include the ALS presented here as well as thermal infrared^18^ and visible range RGB^19^ imagery. Another application of the GNSS/INS data is given in Fig. 5, where the helicopter’s true heading is related to the elevation offset.

### Elevation point clouds

The point cloud data are stored in custom binary format that can be read using the module _bindata of the open-source python package awi_als_toolbox. The point clouds contain elevation values that have not been corrected for atmospheric backscatter, ice drift, or undulations in the elevation. If desired, the user can apply these filters that are part of the filter module of the python package awi_als_toolbox together with the auxiliary information of each flight. In addition, a freeboard variable can be added to the elevation point cloud using the freeboard module and the list of open water points that is part of the auxiliary data of the gridded 30-s segments.

The elevation point clouds offer the highest spatial resolution from the different elevation data products that are part of this publication. With the data being structured in across-track (laser shots per line) and along-track (scan lines) dimensions, various analysis methods can be applied that have been developed in the past for transect surveys and for single-beam laser profilers. The computation of surface roughness presented in Fig. 8 is one example of this. The binary data format also includes information about the helicopter’s position and attitude for each line, which for instance allows to reconstruct the incidence angle of the laser shot as done in the open water detection presented in Fig. 4. The combination of incidence angles, the surface topography, and reflectance measurements could also be used to derive angular dependent emissivities of different surface types.

### Gridded elevation data

The gridded data, both 30-s segments and merged floe grids, are provided in netCDF4 format and do not need specific software to be read. The individual variables of the merged floe grids in GeoTIFF format can be easily visualized and analyzed in any geographic information system software such as ArcGIS or QGIS. In contrast to the elevation point clouds, filters and corrections outlined in Fig. 2b,c have been applied. These data sets also include a freeboard (estimate) variable except for the RTNav transect flights (note the following section on freeboard data quality).

**Fig. 11:**
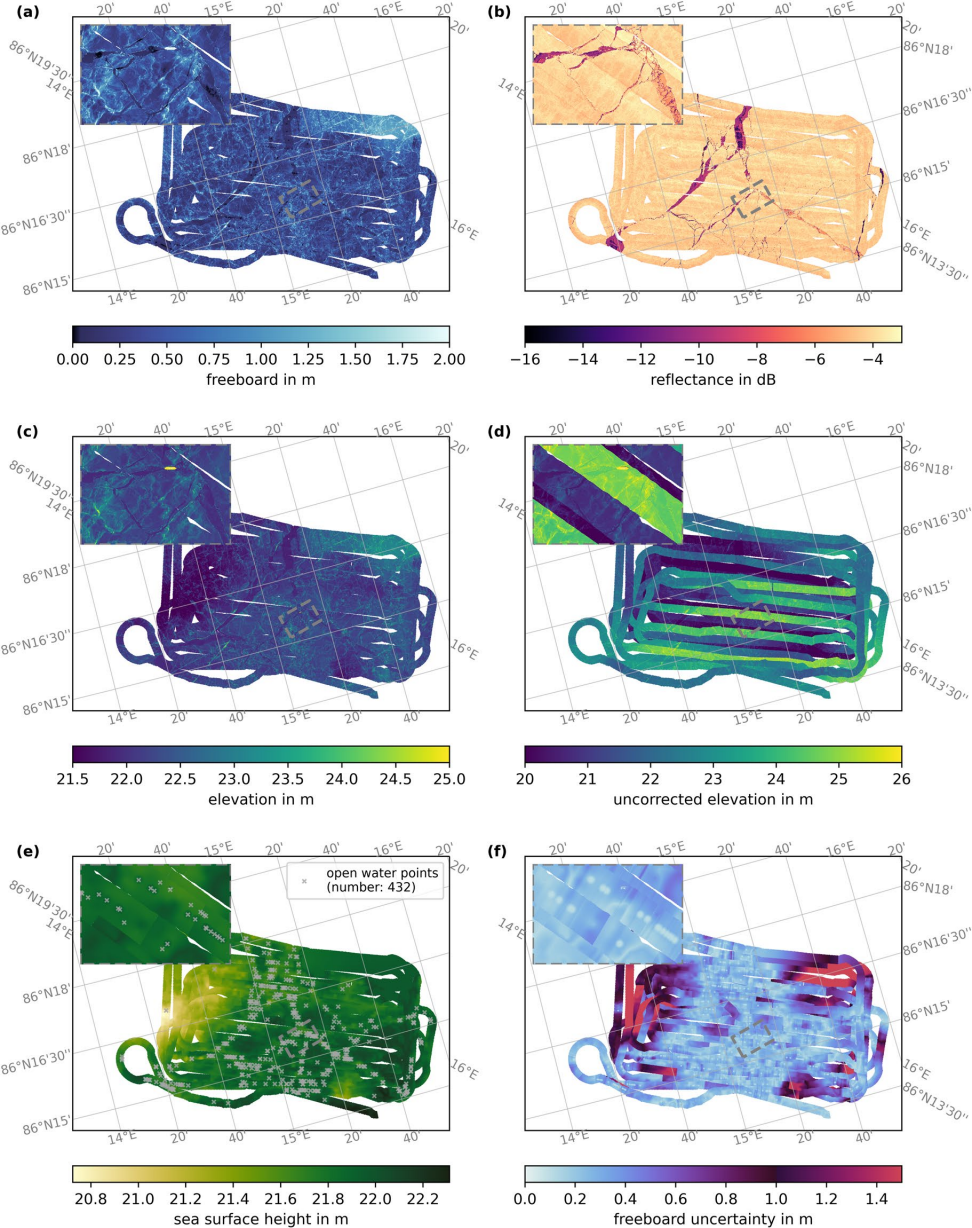
Overview of the ALS data for the floe grid flight from March 21st, 2020. **(a)** shows the freeboard, **(b)** the reflectance, **(c)** the corrected elevation, **(d)** the uncorrected elevation, **(e)** the interpolated and limited sea surface height for the freeboard conversion, and **(f)** the freeboard uncertainty. Detected open water points are marked as gray crosses in **(e)**. The inset in the upper left of each plot highlights the area around the MOSAiC Central Observatory, the position of which is marked with the gray dashed rectangle. For this floe grid flight, only GNSS data with real-time navigation quality is available, which leads to the large discontinuities in the uncorrected elevation. That and an uneven distribution of open water points lead to locally large uncertainty in freeboard.

**Fig. 12:**
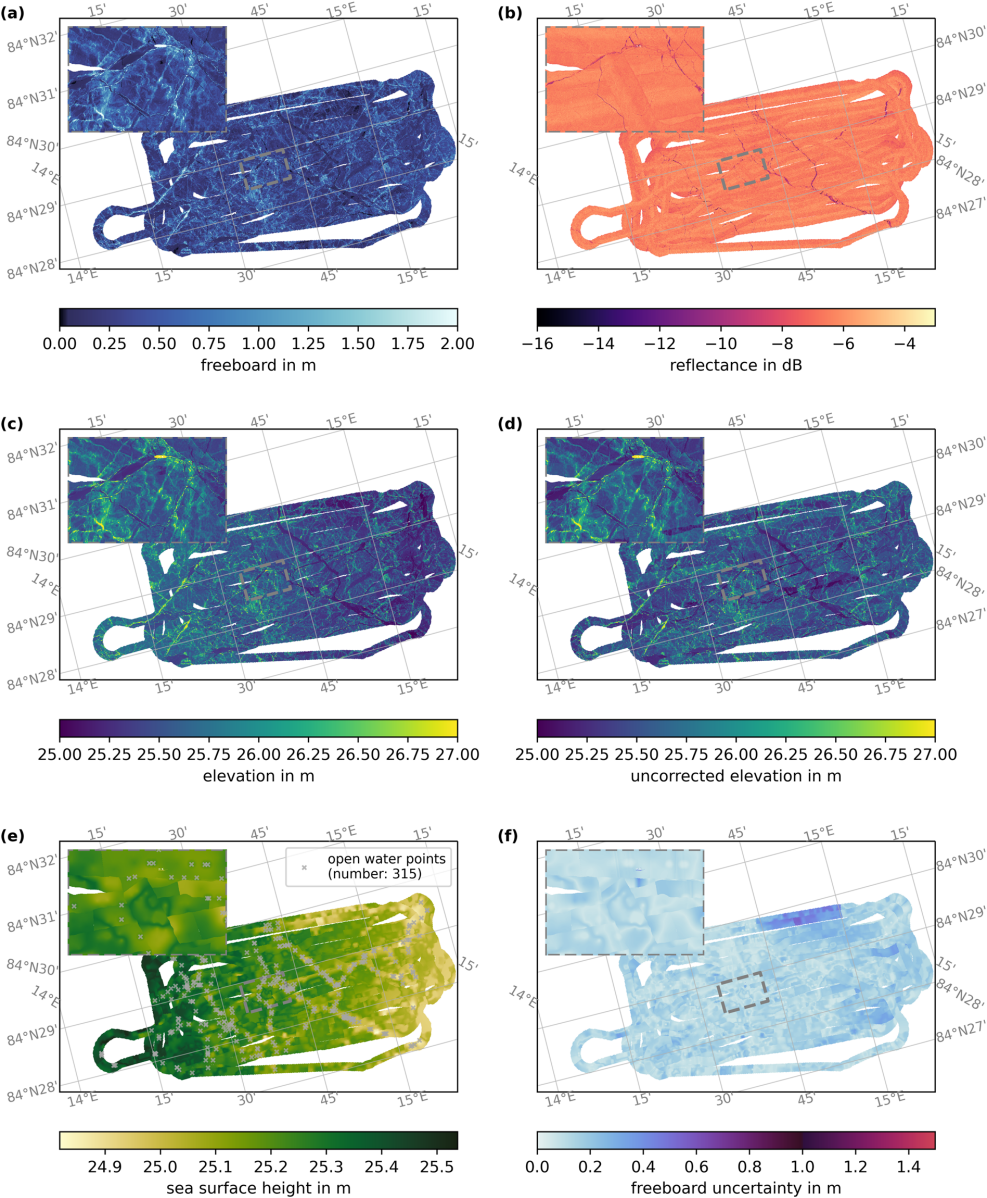
Same as Fig. 11 but for the floe grid flight from April 8th, 2020. For this survey, the GNSS data has precise point positioning quality.

The gridded elevation and freeboard data are of great use for various applications: During the MOSAiC expedition, gridded elevation data from the ALS surveys were a valuable resource in planning and coordinating field activity in particular in the absence of visual photography during the polar night (examples for CO in the insets in Figs. 11 and 12). After the expedition, numerous publications have used prereleased gridded elevation data^37^ or quickview gridded elevations produced on board of Polarstern in near-real-time to visualize the sea-ice conditions during the campaign. Furthermore, first studies have already used prereleased elevation data in their scientific analysis. Webster *et al*.^38^ computed surface roughness from the elevation data and studied its relationship to melt pond formation. Smith *et al*.^39^ used the gridded freeboard data to study the effect of sea-ice topography on the distribution of false bottoms, a thin ice layer beneath fresh-water lenses under the sea ice. Ricker *et al*.^36^ compared gridded elevation of the March 23rd, 2020 survey, a coincident underflight of ICESat-2, with the ATL07 surface elevation product from ICESat-2 to assess the capabilities and uncertainties of ICESat-2 to map small-scale sea-ice features. In addition, Neckel *et al*.^19^ used the ALS reflectance data to correct for cloud shadows in the orthomosaics of the simultaneously acquired RGB data.

### Summary of data uncertainties

In Table 7, we summarize all uncertainty and error estimates discussed above that are relevant to the final data products. Besides the initial instrument uncertainties of the ALS and the INS/GNSS unit, additional uncertainties arise in the processing. We computed uncertainty estimates of the individual processing steps whenever applicable data are available. For instance, the median elevation offset correction applied to floe grids is 0.1 m for PPP and 0.88 m for RTNav. We estimate that in proximity to leads the elevation uncertainty is reduced by these amounts. On the other hand, we can not assess the position uncertainty originating from the ice drift correction, as we do not know the ice deformation during the flight for the survey area. However, we estimate this error to be small to negligible as it is unlikely that major deformation events during the survey flights were not recorded by the MOSAiC team. In addition to the uncertainties summarized in Table 7, we provide grid point estimates of freeboard uncertainty that are described in-depth above in the published data products.

**Table 7:** Summary of all uncertainties discussed in the text sorted by data product, GNSS quality, and flight type.

GNSS quality	Data product	Survey type	Position uncertainty	Altitude uncertainty	Laser accuracy	Elevation uncertainty	Freeboard uncertainty
**PPP**	GNSS/INS data	both	0.05 m	0.1 m	0.025 m	—	—
	Point cloud data	both					
	Gridded elevation data	transect		—		0.125 m	grid point estimates provided
		floe grid	0.05 m + ice drift correction			0.025-0.125 m	
**RTNav**	GNSS/INS data	both	0.46 m	0.91 m		—	—
	Point cloud data	both					
	Gridded elevation data	transect		—		0.935 m	grid point estimates provided
		floe grid	0.46 m + ice drift correction			0.055-0.0935 m	

PPP stands for precise point positioning and RTNav for real-time navigation solutions.

### Note on degraded GNSS altitude quality in winter time

Poor RTNav GNSS quality causes unrealistic undulations in the retrieved surface elevations and impacts the quality of the freeboard. In the section Technical Validation, we highlight at which spatial and temporal scales these undulations occur. For the floe grid flights, the undulations can be mostly filtered using swath-to-swath corrections. For the transect flights, where no correction can be applied due to too little overlap between swaths, we observe large-scale variability in elevation that leads to unrealistic freeboard values in regions distant to open water points. Therefore, we do not publish freeboard data for RTNav transect flights. We provide, however, the detected open-water points for these flights that can be used to convert elevation to freeboard with the methods outlined above. For the RTNav floe grid flights, we provide freeboard with the variable name freeboard_estimate to highlight the possibility of degraded freeboard quality compared to the PPP flights. We recommend all users to assess the elevation and freeboard quality for RTNav surveys in the light of their application, especially for regions distant from leads or if the temporal evolution of point measurements is of interest. We designed an overview plot for each flight that shows the mapped sea-ice freeboard, detected open water points, the uncorrected elevations, and the elevation offset correction term, which was computed for floe grid flights to filter elevation undulations. These overview plots can be used to get an impression of the data quality of each flight without loading the entire data set.

## Data Availability

All codes used to process the data sets are publicly available. Here, we list the repositories used for different processing steps following the order of the flow chart (Fig. 2). Preprocessing and elevation retrieval (Interactive Data Language, IDL): • Converting POSPac MMS 8 output to separate GNSS and INS files: https://gitlab.awi.de/als-seaice/sea-convert • Retrieving ellipsoidal elevation point clouds from the ALS data: https://gitlab.awi.de/als-seaice/als_level1b_seaice Processing gridded data (python3): • Gridding of ALS point cloud data to regular grid including all subroutines presented in Fig. 2b,c: https://github.com/awi-als-toolbox/awi-als-toolbox^40^ • Ice drift correction: https://gitlab.awi.de/floenavi-crs/icedrift • Retrieve position of Polarstern: https://gitlab.awi.de/floenavi-crs/floenavi General processing scripts (bash, python3): • Batch processing scripts and config files with flight-specific parameters https://gitlab.awi.de/als-seaice/mosaic-als-proc
